# Factors associated with healthcare utilization among community-dwelling elderly in Shanghai, China

**DOI:** 10.1371/journal.pone.0207646

**Published:** 2018-12-03

**Authors:** Man Jiang, Guang Yang, Lvying Fang, Jin Wan, Yinghua Yang, Ying Wang

**Affiliations:** 1 School of Public Health, Fudan University, Shanghai, China; 2 Eye & ENT Hospital of Fudan University, Shanghai, China; 3 Ruijin Hospital, Shanghai Jiao Tong University School of Medicine, Shanghai, China; 4 Management Department, Shanghai Municipal Center For Disease Control & Prevention, Shanghai, China; 5 School of Public Health/Key Lab of Health Technology Assessment, National Health and Family Planning Commission of the People's Republic of China, Fudan University, Shanghai, China; The Ohio State University, UNITED STATES

## Abstract

**Objective:**

The objective of this study was to evaluate the factors associated with the health status of older Chinese people living in the community, in order to inform strategies to expand access to healthcare.

**Methods:**

Two-phase stratified cluster sampling was applied; 2000 older people participated in this study. Face-to-face interviews were conducted in Shanghai between June and August, 2011. Descriptive analysis was used to examine the respondents’ characteristics. Based on Andersen’s healthcare utilization model, a chi-squared test and multiple logistic regression were performed to examine the influences of predisposing, enabling, need, and contextual factors on healthcare utilization.

**Results:**

We found that 44.5% of the older people in the sample had good self-reported health status, while 12.8% were poor, 14.5% had visited hospitals or clinics as outpatients in the previous two weeks, and 16.5% had been hospitalized in the previous year. Logistic regression analysis revealed that outpatient health services were more likely to be used by women and those whose income was from friends or social relief, who had poor to good self-reported health status, who were experiencing declining health, who engaged in volunteer activities, and who had chronic diseases. Meanwhile, hospitalization was more likely among those in the older age groups, those with pension income, living in outer suburbs, with poor self-reported health status, experiencing difficulty with activities of daily living and outdoor activities, or having a chronic disease.

**Conclusions:**

The results showed the impact of economic status, health status, demographic and social characteristics, and other factors on the health service utilization of elderly people living in the community in Shanghai. Need variables were the strongest predictors of health service use, although contextual factors also contributed.

## Introduction

An aging society is one where more than 10% of the population is over 60 years old and/or 7% are over 65 [1]. According to statistics published by the WHO, the percentage of the global population aged 60 and over was 11% by the end of 2011, while that in China was 13% [2]. China is a therefore recognized as an aging society, with Shanghai showing a more extreme position. According to the Shanghai Bureau of Statistics, the city had a population of 14.50 million people registered as living in households by the end of 2016, of whom 31.59% were aged 60 and over, with this percentage increasing annually [3].

This rapidly aging population poses significant challenges for healthcare [4]. With their declining physical function and increasing morbidity from various diseases, the demand for healthcare services from older people is far higher than from other age groups [5]. For instance, 33% of healthcare expenditure in the United States is spent on older people [6]. There is growing recognition globally of the need to evaluate how healthcare services are utilized, and how healthcare systems might best be enhanced to meet the health needs of an aging population [7].

Healthcare utilization means obtaining healthcare from health service providers [8]. Many theoretical models of healthcare utilization have been formulated, interpreting it from various perspectives (such as economic, psychosocial, behavioral, and epidemiological) and exploring which variables influence it and to what degree [9]. For example, the Andersen–Newman model [10] explains healthcare utilization in terms of relationships among predisposing, enabling, need, and contextual factors found in the general population, while Berki and Kobashigawa [11] emphasized the importance of services, socioeconomic factors, and individual characteristics. Other studies focused on vulnerable populations, for example, minority groups or immigrants. Mutchler and Burr [12] examined racial differences in health service utilization, and Aroian et al. [13] focused on elderly immigrants from the former Soviet Union. Factors associated with healthcare utilization can be divided into three types [14]: physiological (e.g., sex, age, race, health status), social (e.g., income, education, social status), and subjective (e.g., self-reported health status).

China is the developing country with the largest elderly population, partly as a result of the implementation of its “One Child” policy in the 1970s [15]. Along with the aging trend, China is experiencing a significant health transition, with older people generally living longer generally but also with increasing years in suboptimal perceived health accompanied by chronic diseases [16]. The problem of healthcare utilization has been studied by some investigators in China, but these studies have not properly considered influencing factors, contextual factors, or disease status. Andersen’s model is a useful framework for studying health service use and for grouping the factors shown to affect health service utilization in older Chinese people [17]. Uncovering factors associated with health service use is important, particularly when used concurrently with conventional care, as this could help avoid potential problems.

Shanghai was used as the study area, because it has the most severe aging situation in China [18].We examined how predisposing, enabling, need, and contextual factors were related to healthcare utilization. Outpatient service usage rates in the previous two weeks and hospital inpatient services in the previous year were set as dependent variables [19]. The objective of the study was to evaluate factors influencing health status and healthcare utilization among older Chinese people, gathering reference data for policies to improve the healthcare accessibility for the elderly and for the development of health management and healthy aging programs for older people in China and other developing countries with similarly aging populations.

### Theoretical framework

First developed in the late 1960s, Andersen’s healthcare utilization model was originally used to measure equitable access to health services and assist in developing policies to promote such access. It aimed to integrate several ideas about how and why health services were used [20], and has been widely used to explore relationships between predisposing, enabling, and need factors and healthcare utilization [21] in a wide variety of contexts, for example predicting emergency room use [22, 23] and patient satisfaction [24].

Predisposing factors are those increasing individuals’ propensity to use services; they include demographic and social characteristics such as sex, age, marital status, race, education level, children, and living conditions. Enabling factors increase individual ability to access services, and includes family and social resources, health insurance, pension or other income, and living location. Need factors reflect illness level and factors affecting it, including self-reported health status, sensory damage, loneliness, ability to perform activities of daily living (ADLs), changes in health status, smoking and alcohol-drinking status, and presence of a certain chronic disease. The need component involves both health professionals’ and individuals’ perceptions of whether clinical factors require use of healthcare services.

Previous studies have shown that the strongest predictors of healthcare utilization are need factors, followed by enabling and predisposing factors [25]. Some studies have also shown that contextual factors play key roles; for example, geographic variations influence length of hospital stay [26, 27]. Neighborhood [28], characteristics of providers [29] and social capital–related factors such as social trust, civic engagement, and social relations [30] all affect health service utilization. Unlike other age groups, the high incidence of chronic diseases among older people will lead to changes in their health service utilization. Many elderly people have multiple concurrent prevalent diseases at the same time, while most previous studies only considered if people had any chronic diseases or not (yes/no), rather than explore the impact of each disease [17, 31]. It has therefore been necessary to evaluate healthcare utilization using a specialized version of Andersen’s model.

Our study extends Andersen’s model to include the most prevalent diseases in this population as special need factors as well as contextual factors, and aims to determine whether these special variables add predictability to health service utilization. The most prevalent diseases, which can be analyzed as a separate part of the need factors, include hypertension, heart disease, diabetes, cataracts, cerebrovascular disease, bronchitis, and gastroenteritis. Contextual factors considered here include regional economic development, participation in outdoor and community activities, and participation in volunteer work.

## Materials and methods

### Design and procedures

The phrase “older people” in China generally applies to those who are 60 years old and over; we therefore focused on people aged above 60 living in communities served by the sample community institutions.

We decided on a stratified random sample, and the effect size was estimated as two, meaning that the sample size required was doubled. We estimated a 15% loss to follow-up, so a sample of 1756 older people was needed. We eventually received 2000 valid questionnaires. Ethical approval was received from the Fudan University Research Ethics Committee. Respondents were assured that participation in the study was voluntary, with the return of completed questionnaires being taken as consent; the study data of respondents were collected anonymously.

A cross-sectional design was used to investigate these community-dwelling older Shanghainese adults, in August 2011. The 18 districts (counties) of Shanghai were divided into three levels stratified by socioeconomic status: high, medium, and low. Random sampling was conducted for two districts from each level, with samples collected on the basis of population size. High-SES districts were Pudong (sample of 832) and Changning (199); medium-SES ones were Hongkou (291) and Putuo (274); low-SES ones were Jinshan (157) and Chongming (248). We then randomly selected one street or town (local center) in the medium-SES districts, arranged all their residents in alphabetical order by name, and surveyed them one by one until we had a large enough sample.

The study design and questionnaire were created by the School of Public Health at Fudan University and piloted in 200 elderly people, and then revised. Face-to-face interviews were conducted in June and August 2011. The sampled communities were responsible for coordination with the interviewees and training the investigators. All the interviewers, who included research assistants and experienced peer fieldworkers, had received extensive training on research ethics and assessment methodology prior to data collection. A small gift equivalent in value to US$3, was given to the participants as a token of appreciation for their participation.

### Variable content

Adequate operationalization and selection of variables representing the Andersen model was ensured by considering Andersen’s own suggestions [20] as well as known information on the relationships between various factors and health service utilization among the middle-aged and elderly in China, an approach again largely based on the framework of Andersen’s behavioral model [31, 32]. In this study, healthcare utilization was quantified by assessing (1) level of use of outpatient care in the previous two weeks, including family doctor, nursing or specialist visits, and (2) hospitalization(s) in the last year.

#### Predisposing factors

Socio-demographic data gathered included age, gender, education, marital status, nationality, number of children, living situation, and healthy lifestyle. Age was divided into five groups: 60–64, 65–69, 70–74, 75–79, and ≥80. Three marital statuses were used: married, separated/divorced, widowed. Education had four categories: (1) illiterate, including semi-literate, less than primary education, or home study; (2) primary education; (3) secondary education, including middle and high school as well as vocational education; and (4) higher education, including associate’s, bachelor’s, master’s, and doctoral degrees. Living situation was divided into three types: living alone, living with spouse, and living with children. Healthy lifestyles, which serve as a proxy for health beliefs, were measured by two variables: (1) never smoke, smoke at times, smoke often, or had quit smoking; (2) never drink, drink at times, often drink, or had quit drinking alcohol.

#### Enabling factors

The enabling factors in the model include healthcare insurance, pension income, source of income, and location. China’s basic medical insurance system can be divided into three types: medical insurance for urban employees, medical insurance for urban and town residents, and “new-type rural cooperative medical scheme” (NRCMS). In addition to these three basic types, we also investigated the proportion of elderly whose healthcare expenses are self-paid or publicly funded. Pension income and source of income can also help capture the accessibility of health services from an economic perspective. In addition, people living in different locations—city center, inner suburbs, and outer suburbs—have different degrees of access to transportation and medical facilities.

#### Need factors

The need factors in the model include self-reported health, sensation disorders, feeling lonely or nervous, activity of daily living (ADL) limitations, and chronic diseases. Self-reported health is based on the respondents’ answer to the questions “Would you say your health is good, normal, or poor?” and “Compared with last year, what changes have you made in your health?” Three questions related to mental health, “Do you have sensation disorders?” (yes/no) and “Do you feel lonely or nervous?” (never/sometimes/always), were also included in the questionnaire. Functional ability was assessed using the Barthel Index, which has been regarded as the best tool for this purpose in terms of sensitivity, simplicity, communicability, scalability, and ease of scoring [33]. First published in 1965, its ten items cover eating, dressing and undressing, making up, walking, getting into and out of bed, washing and bathing, going up and down stairs, and toileting and controlling bladder and bowel movements [34]. Comorbidity was measured as the self-reported number of chronic diseases that had been diagnosed by a physician, coded into categories of hypertension, diabetes, cataract, cerebrovascular disease, bronchitis, gastroenteritis, intervertebral disc disease, cardiovascular disease, and asthma.

### Data analysis

SPSS Statistics for Windows (version 20.0; IBM Corp., Armonk, NY, USA) was used to analyze the data. Mean and standard deviation were used in the descriptive statistics. The chi-squared test was used to determine the differences between socio-demographic characteristics. The significance threshold was P < 0.05.

The relationships among socio-demographic characteristics, living habits, social support, mental and physical status, and self-reported health status were tested by the chi-squared test. A series of logistic regression models were performed to establish the independent associations between health service utilization and its determinants. The predictors in Model 1 were based on Andersen’s model; Model 2 tested whether the addition of contextual factors adds incremental predictive power; and Model 3 tested whether the addition of disease status adds incremental predictive power. The index of -2Log Likelihood was used to compare model fit of different models [35]. A p-value less than 0.05 was considered statistically significant.

## Results

### Socio-demographic characteristics

The total sample size comprised 2000 older people. The response rate was 100%, with 57.8% being women. The mean age was 71.61 years, and the proportions in each age group (aged 60–64, 65–69, 70–74, 75–79 and ≥80) were around 2:1:1:1:1. The predominant nationality of most was Han (98.9%), with 1.1% being ethnic minorities; 75.8% were married, 21.6% were widowed, and 2.6% were divorced or single. In all, 28.7% had received no formal education. Most lived with a spouse (86.0%), although 35.0% lived with children, and 14.0% lived alone. Medical insurance coverage was good, with 31.5% being part of a medical insurance system for urban and town residents, 45.5% one for urban employees, and 14.7% an NRCMS. Finally, 41.6% lived in the inner suburbs, 38.2% in the city center, and 20.2% in the outer suburbs.

### Health status and healthcare utilization

During the previous two weeks, 380 had been ill and 1620 had not. The two-week prevalence of illness was 19.0%, and the two-week visit rate to outpatient services was 14.5%. The rate of not seeking medical care by patients who had been ill in the previous two weeks was 23.9%, while the hospitalization rate in the previous year was 16.5%. Overall, 44.5% reported good health status, 42.8% normal, and 12.8% poor health status. Most, 83.0%, reported that they did not feel lonely, and 89.2% were not nervous; 54.7% felt satisfied with life, and 5.1% were not. Finally, 77.2% had at least one chronic disease.

### Univariate analysis of outpatients’ health service utilization

Table 1 shows the chi-squared test results for each Andersen model predictor of two-week visit rate. Of the predisposing predictors, only gender and previously having smoked had any relationship to outpatient health service utilization: men were less likely to use outpatient health services than women. Three of the enabling predictors were found related to outpatient health service use: pension income level, source of income, and location. Respondents with pension income of 1000–2000 RMB monthly, whose income source was a pension, and who lived in the outer suburbs were more likely to use outpatient health services. Need predictor characteristics related to outpatient health service use were poor self-reported health status, sensation disorders, feeling lonely and/or nervous, poor satisfaction with life, limitation to activities of daily living (ADLs), health status changing for the worse, and having a chronic disease. Respondents with chronic diseases such as heart disease, cataracts, cerebrovascular disease, and gastroenteritis were particularly more likely to use outpatient health services. Living in a poorer region and having more contact with friends and neighbors were also related to outpatient health service use.

**Table 1:** Univariate analysis of outpatient health service utilization.

Variable	Yes		No		Sum		Two-week visit rate	
	#	%	#	%	#	%	χ^2^	p
**Predisposing factors**								
**Gender**							9.55	0.002
male	98	11.6	745	88.4	843	42.2		
female	191	16.6	963	83.4	1154	57.8		
**Age group (years)**							3.456	0.485
60–64	88	13.7	555	86.3	643	32.2		
65–69	46	13.6	293	86.4	339	17.0		
70–74	54	17.2	260	82.8	314	15.7		
75–79	59	15.6	319	84.4	378	18.9		
≥80	42	12.9	283	87.1	325	16.3		
**Marital status**							0.231	0.891
widowed	59	13.7	372	86.3	431	21.6		
divorced/single	8	15.7	43	84.3	51	2.6		
married	218	14.4	1293	85.6	1511	75.8		
**Nationality**							1.618	0.203
Han nationality	288	14.6	1687	85.4	1975	98.9		
ethnic minority	1	4.8	20	95.2	21	1.1		
**Education level**							1.645	0.649
illiterate	80	14.0	492	86.0	572	28.7		
primary education	96	15.1	540	84.9	636	31.9		
secondary education	91	13.5	581	86.5	672	33.7		
higher education	20	17.5	94	82.5	114	5.7		
**Number of children**							1.026	0.795
0	3	11.5	23	88.5	26	1.3		
1 or 2	153	14.5	904	85.5	1057	53.1		
3 or 4	106	14.0	652	86.0	758	38.1		
5 or more	25	16.9	123	83.1	148	7.4		
**Living situation**							0.608	0.738
living alone	44	15.9	233	84.1	277	14.0		
living with spouse	142	14.1	868	85.9	1010	51.0		
living with children	102	14.7	591	85.3	693	35.0		
**Healthy lifestyle**								
**Smoking**							8.491	0.037
never	237	15.2	1321	84.8	1558	77.9		
at times	5	6.0	79	94.0	84	4.2		
often	28	11.2	221	88.8	249	12.5		
quit	19	17.4	90	82.6	109	5.5		
**Drinking**							3.638	0.303
never	238	15.2	1329	84.8	1567	78.4		
at times	17	11.3	133	88.7	150	7.5		
often	5	9.3	49	9.7	54	2.7		
quit	29	12.7	200	87.3	229	11.5		
**Enabling factors**								
**Healthcare insurance**							1.783	0.776
for urban employees	87	14.0	533	86.0	620	31.5		
for urban and town residents	132	14.8	762	85.2	894	45.5		
NRCMS	44	15.2	245	84.8	289	14.7		
at own expenses	2	7.7	24	92.3	26	1.3		
at public expense	23	16.8	114	83.2	137	7.0		
**Pension income level (RMB)**							6.723	0.035
0–999	147	14.8	847	85.2	994	49.9		
1000–1999	73	17.5	344	82.5	417	20.9		
2000+	68	11.7	512	88.3	580	29.1		
**Source of income**							52.925	0.000
pension	230	13.6	1464	86.4	1694	86.1		
work or savings	17	11.8	127	88.2	144	7.3		
family	5	8.9	51	91.1	56	2.8		
others	32	43.2	42	56.8	74	3.8		
**Location**							9.646	0.008
city center	114	14.9	650	85.1	764	38.2		
inner suburbs	100	12.0	732	88.0	832	41.6		
outer suburbs	75	18.6	329	81.4	404	20.2		
**Need factors**								
**Self-reported health status**							92.8	0.000
good	76	8.6	812	91.4	888	44.5		
normal	130	15.2	724	84.8	854	42.8		
poor	83	32.5	172	67.5	255	12.8		
**Sensation disorders**							7.010	0.008
no	140	12.6	972	87.4	1112	55.6		
yes	149	16.8	739	83.2	888	44.4		
**Feeling lonely**							12.403	0.002
never	220	13.3	1439	86.7	1659	83.0		
sometimes	57	21.3	210	78.7	267	13.4		
always	12	16.4	61	83.6	73	3.7		
**Feeling nervous**							8.175	0.017
never	244	13.7	1539	86.3	1783	89.2		
sometimes	37	21.1	138	78.9	175	8.8		
always	8	20.0	32	80.0	40	2.0		
**Life satisfaction**							32.98	0.000
good	121	11.1	971	88.9	1092	54.7		
fair	137	17.1	666	82.9	803	40.2		
poor	30	29.4	72	70.6	102	5.1		
**ADLs**							7.120	0.008
independent	278	14.2	1685	85.8	1963	98.2		
dependent for ≥1 activity	11	29.7	26	70.3	37	1.8		
**Physical health change**							81.439	0.000
better	8	8.1	91	91.9	99	5.0		
unchanged	124	9.7	1153	9.3	1277	64.0		
worse	145	24.9	437	75.1	582	29.2		
unstable	9	24.3	28	75.7	37	1.9		
**With chronic disease**							21.929	0.000
no	35	7.7	421	92.3	456	22.8		
yes	254	16.5	1290	83.5	1544	77.2		
**Number of chronic diseases/person**							81.046	0.000
0	35	7.7	421	92.3	456	22.8		
1	79	11.7	596	88.3	675	33.8		
2	67	15.4	369	84.6	436	21.8		
3	46	18.3	205	81.7	251	12.6		
4 or more	62	34.1	120	65.9	182	9.1		
**Disease status**								
**Hypertension**							2.457	0.117
yes	160	15.7	862	84.3	1022	51.1		
no	129	13.2	849	86.8	978	48.9		
**Heart diseases**							34.256	0.000
yes	101	23.2	335	76.8	436	21.8		
no	188	12.0	1376	88.0	1564	78.2		
**Diabetes**							2.699	0.100
yes	51	17.6	239	82.4	290	14.5		
no	238	13.9	1472	86.1	1710	85.5		
**Cataract**							11.24	0.001
yes	40	23.0	134	77.0	174	8.7		
no	249	13.6	1577	86.4	1826	91.3		
**Cerebrovascular disease**							5.26	0.022
yes	31	20.8	118	79.2	149	7.4		
no	258	13.9	1593	86.1	1851	92.6		
**Bronchitis**							2.297	0.130
yes	23	19.2	97	80.8	120	6.0		
no	266	14.1	1614	85.9	1880	94.0		
**Gastroenteritis**							38.999	0.000
yes	39	34.5	74	65.5	113	5.65		
no	250	13.2	1637	86.8	1887	94.35		
**Contextual factors**								
**Regional economic level**							11.987	0.002
good	123	11.9	907	88.1	1030	51.5		
middle	91	16.1	475	83.9	566	28.3		
poor	75	18.6	329	81.4	404	20.2		
**Outdoor activities**							0.166	0.683
yes	165	14.2	1001	85.8	1166	58.4		
no	123	14.8	708	85.2	831	41.6		
**Seeing children**							3.050	0.550
every day	174	13.6	1109	86.4	1283	65.2		
every week	64	15.6	346	84.4	410	20.8		
every month	32	16.5	162	83.5	194	9.9		
every year	11	16.7	55	83.3	66	3.4		
<1 time/year	1	6.2	15	93.8	16	0.8		
**Neighbor contact**							0.317	0.957
every week	259	14.4	1537	85.6	1796	89.8		
every month	11	14.5	65	85.5	76	3.8		
every year	3	18.8	13	81.2	16	.8		
almost never	15	13.5	96	86.5	111	5.6		
**Gathering with relatives**							5.034	0.169
every week	59	13.2	389	86.8	448	22.4		
every month	50	16.0	262	84.0	312	15.6		
every year	137	15.7	735	84.3	872	43.6		
almost never	42	11.4	325	88.6	367	18.4		
**Community activities**							19.496	0.000
every week	67	22.2	235	77.8	302	15.2		
every month	15	9.7	140	9.3	155	7.8		
every year	24	11.5	184	88.5	208	10.4		
almost never	182	13.7	1146	86.3	1328	66.6		
**Volunteer activities**							1.951	0.162
yes	45	17.4	214	82.6	259	13.0		
no	244	14.1	1487	85.9	1731	87.0		

### Logistic regression analysis of outpatient healthcare services utilization

The inclusion level was set to p < 0.05 and the exclusion criterion to p > 0.1. Then, all the variables were included in stepwise regression; only the variables in the final results are shown. Table 2 shows the logistic regression analysis results of each Andersen model predictor of outpatient visit rate in the previous two weeks, as the dependent variable. In all three models, gender (model 1: OR 1.344; 95% 0.994–1.818, p = 0.064) was not statistically significant. Compared to those whose income was from a pension, those who had income from other sources (model 1: OR 6.497; 95% 3.599–11.727, p = 0.000) were more likely to use outpatient healthcare services. The statistically significant need predictors were poor self-reported health status (model 1: OR 6.497; 95% 3.599–11.727, p = 0.000), normal satisfaction with life (model 1: OR 1.472; 95% 1.088–1.992, p = 0.012), and a change for the worse in physical health (model 1: OR 3.301; 95% 1.502–7.258, p = 0.003). As for contextual factors, elderly who engaged in volunteering (no vs. yes) (model 3: OR 0.619; 95% 0.415–0.924, p = 0.019) were more likely to use health services. Of the newly added disease factors in Model 3, both heart diseases (model 3: OR 1.693; 95% 1.234–2.324, p = 0.001) and gastroenteritis (model 3: OR 2.181; 95% 1.315–3.616, p = 0.003) were associated with the utilization of health services.

**Table 2:** Logistic regression analysis of outpatient healthcare services utilization.

Variable	Model 1		Model 2		Model 3	
	Sig.	OR (95%CI)	Sig.	OR (95%CI)	Sig.	OR (95%CI)
**Predisposing factors**						
**Gender** (female vs. male)	0.055	1.344 (0.994–1.818)	0.064	1.33 (0.983–1.8)	0.135	1.263 (0.93–1.715)
**Enabling factors**						
**Pension income level (RMB)**						
0–999	ref		ref		ref	
1000–1999	0.104	1.346 (0.941–1.924)	0.147	1.305 (0.911–1.869)	0.092	1.367 (0.95–1.967)
2000+	0.280	0.812 (0.557–1.185)	0.222	0.79 (0.541–1.153)	0.232	0.791 (0.539–1.161)
**Source of income**						
pension	ref		ref		ref	
work or savings	0.315	0.731 (0.397–1.347)	0.355	0.75 (0.408–1.38)	0.333	0.74 (0.402–1.362)
family	0.087*	0.422 (0.157–1.135)	0.100	0.437 (0.163–1.173)	0.182	0.508 (0.188–1.372)
others	0.000*	6.497 (3.599–11.727)	0.000*	6.644 (3.669–12.03)	0.000*	7.322 (4.031–13.3)
**Need factors**						
**Self-reported health status**						
good	ref		ref		ref	
normal	0.116	1.311 (0.935–1.837)	0.079	1.356 (0.966–1.904)	0.179	1.265 (0.898–1.782)
poor	0.000*	2.747 (1.78–4.24)	0.000*	2.923 (1.886–4.53)	0.000*	2.469 (1.572–3.877)
**Life satisfaction**						
good	ref		ref		ref	
normal	0.012*	1.472 (1.088–1.992)	0.010*	1.492 (1.101–2.021)	0.014*	1.47 (1.083–1.997)
poor	0.146	1.525 (0.864–2.693)	0.144	1.53 (0.865–2.705)	0.340	1.333 (0.739–2.403)
**Physical health change**						
better	ref		ref		ref	
unchanged	0.415	1.385 (0.633–3.027)	0.404	1.395 (0.639–3.049)	0.321	1.496 (0.675–3.313)
worse	0.003	3.301 (1.502–7.258)	0.003*	3.351 (1.524–7.367)	0.003*	3.344 (1.5–7.453)
unstable	0.005*	4.797 (1.587–14.49)	0.006*	4.719 (1.559–14.284)	0.008*	4.576 (1.494–14.011)
**Disease status**						
**Heart diseases** (yes vs. no)					0.001*	1.693 (1.234–2.324)
**Gastroenteritis **(yes vs. no)					0.003*	2.181 (1.315–3.616)
**Contextual Factors**						
**Volunteer activities **(no vs. yes)			0.012 *	0.603 (0.407–0.894)	0.019*	0.619 (0.415–0.924)
**Chi-squared**	166.366		172.327		192.011	
**df**	13		14		16	
**Sig.**	0.000		0.000		0.000	
**-2Log Likelihood**		1339.348	1333.388		1313.703	

*p < 0.05;

CI: confidence interval.

The index of -2Log Likelihood was 1339.348 for model 1. After including contextual factors, in model 2, this index dropped to 1333.388. This was further reduced to 1313.703 when disease status was added. Therefore, model 3 was the optimal model.

### Univariate analysis of hospitalization

Table 3 shows the chi-squared test results for each Andersen model predictor of hospitalization rate. Four predisposing factors were related to hospitalization service use: age group, marital status, education and number of children. Older, less educated, and widowed people with more children were more likely to use hospital services. The enabling predictors source of income and region were also related to hospitalization service. Respondents whose income was from work or savings were less likely to have been hospitalized than those whose income was provided by their family. Those living in the outer suburbs were more likely to have been hospitalized. Need predictors related to hospitalization were poor self-reported health status, sensation disorders, feeling lonely or nervous, having poor satisfaction with life, limitation in one or more activities of daily living (ADLs), change for the worse in physical health, previously having smoked, and having one or more chronic diseases.

**Table 3:** Univariate analysis of hospitalization.

	Yes		No			Sum				Hospitalization rate	
Variable	#	%	#		%	#		%		χ^2^	p
**Predisposing factors**											
**Gender**										0	0.988
male	139	16.5	704		83.5	843		42.2			
female	190	16.5	964		83.5	1154		57.8			
**Age group (years)**										45.695	0.000
60–64	65	10.1	578		89.9	643		32.2			
65–69	43	12.7	296		87.3	339		17.0			
70–74	62	19.7	252		80.3	314		15.7			
75–79	82	21.7	296		78.3	378		18.9			
≥80	78	24.0	247		76.0	325		16.3			
**Marital status**										6.929	0.031
widowed	88	20.4	343		79.6	431		21.6			
divorced/single	6	11.8	45		88.2	51		2.6			
married	233	15.4	1278		84.6	1511		75.8			
**Nationality**										0.101	0.750
Han nationality	325	16.5	1650		83.5	1975		98.9			
ethnic minority	4	19.0	17		81.0	21		1.1			
**Education level**										6.761	0.080
illiterate	113	19.8	459		80.2	572		28.7			
primary education	100	15.7	536		84.3	636		31.9			
secondary education	97	14.4	575		85.6	672		33.7			
higher education	19	16.7	95		83.3	114		5.7			
**Number of children**										23.287	0.000
0	2	7.7	24		92.3	26		1.3			
1 or 2	138	13.1	919		86.9	1057		53.1			
3 or 4	156	20.6	602		79.4	758		38.1			
5 or more	33	22.3	115		77.7	148		7.4			
**Living situation**										4.021	0.134
living alone	39	14.1	238		85.9	277		14.0			
living with spouse	160	15.8	850		84.2	1010		51.0			
living with children	130	18.8	563		81.2	693		35.0			
**Healthy lifestyle**											
**Smoking**										18.085	0.000
never	264	16.9	1294		83.1	1558		77.9			
at times	11	13.1	73		86.9	84		4.2			
often	25	10.0	224		90.0	249		12.5			
quit	30	27.5	79		72.5	109		5.5			
**Drinking**										0.695	0.874
never	262	16.7	1305		83.3	1567		78.4			
at times	23	15.3	127		84.7	150		7.5			
often	7	13.0	47		87.0	54		2.7			
quit	38	16.6	191		83.4	229		11.5			
**Enabling factors**											
**Healthcare insurance**										9.201	0.056
for urban employees	85	13.7	535		86.3	620		31.5			
for urban and town residents	147	16.4	747		83.6	894		45.5			
NRCMS	58	20.1	231		79.9	289		14.7			
at own expenses	5	19.2	21		80.8	26		1.3			
at public expense	30	21.9	107		78.1	137		7.0			
**Pension income level (RMB)**										4.773	0.092
0–999	175	17.6	819		82.4	994		49.9			
1000–1999	54	12.9	363		87.1	417		20.9			
2000+	98	16.9	482		83.1	580		29.1			
**Source of income**										8.111	0.044
pension	278	16.4	1416		83.6	1694		86.1			
work or savings	18	12.5	126		87.5	144		7.3			
family	16	28.6	40		71.4	56		2.8			
others	10	13.5	64		86.5	74		3.8			
**Location**										13.186	0.001
city center	122	16.0	642		84.0	764		38.2			
inner suburbs	118	14.2	714		85.8	832		41.6			
outer suburbs	90	22.3	314		77.7	404		20.2			
**Need factors**											
**Self-reported health status**										116.472	0.000
good	83	9.3	805		90.7	888		44.5			
normal	151	17.7	703		82.3	854		42.8			
poor	96	37.6	159		62.4	255		12.8			
**Sensation disorders**										33.141	0.000
no	136	12.2	976		87.8	1112		55.6			
yes	194	21.8	694		78.2	888		44.4			
**Feeling lonely**										25.467	0.000
never	244	14.7	1415		85.3	1659		83.0			
sometimes	72	27.0	195		73.0	267		13.4			
always	14	19.2	59		80.8	73		3.7			
**Feeling nervous**										28.928	0.000
never	267	15.0	1516		85.0	1783		89.2			
sometimes	50	28.6	125		71.4	175		8.8			
always	13	32.5	27		67.5	40		2.0			
**Life satisfaction**										23.205	0.000
good	162	14.8	930		85.2	1092		54.7			
fair	133	16.6	670		83.4	803		40.2			
poor	34	33.3	68		66.7	102		5.1			
**ADLs**											
independent	308	15.7	1655		84.3	1963		98.2		50.496	0.000
dependent for > = 1 activity	22	59.5	15		40.5	37		1.8			
**Physical health change**										105.729	0.000
better	33	33.3	66		66.7	99		5.0			
unchanged	130	10.2	1147		89.8	1277		64.0			
worse	158	27.1	424		72.9	582		29.2			
unstable	7	18.9	30		81.1	37		1.9			
**With chronic disease**										42.198	0.000
no	30	6.6	426		93.4	456		22.8			
yes	300	19.4	1244		80.6	1544		77.2			
**Disease states**											
**Hypertension**										7.932	0.005
yes	192	18.8	830		81.2	1022		51.1			
no	138	14.1	840		85.9	978		48.9			
**Heart diseases**										41.326	0.000
yes	116	26.6	320		73.4	436		21.8			
no	214	13.7	1350		86.3	1564		78.2			
**Diabetes**										4.321	0.038
yes	60	20.7	230		79.3	290		14.5			
no	270	15.8	1440		84.2	1710		85.5			
**Cataracts**										9.33	0.002
yes	43	24.7	131		75.3	174		8.7			
no	287	15.7	1539		84.3	1826		91.3			
**Cerebrovascular disease**										155.849	0.000
yes	79	53.0	70		47.0	149		7.4			
no	251	13.6	1600		86.4	1851		92.6			
**Bronchitis**										34.634	0.000
yes	43	35.8	77		64.2	120		6.0			
no	287	15.3	1593		84.7	1880		94.0			
**Gastroenteritis**										5.958	0.015
yes	28	24.8	85		75.2	113		5.65			
no	302	16.0	1585		84.0	1887		94.35			
**Number of chronic diseases/person**										124.714	0.000
0	30	6.6	426		93.4	456		22.8			
1	78	11.6	597		88.4	675		33.8			
2	89	20.4	347		79.6	436		21.8			
3	64	25.5	187		74.5	251		12.6			
4 or more	69	37.9	113		62.1	182		9.1			
**Two-week outpatient visit**										29.384	0.000
yes	98	25.8	282		74.2	380		19.0			
no	232	14.3	1388		85.7	1620		81.0			
**Contextual factors**											
**Regional economic level**										20.933	0.000
good	134	13.0	896		87.0	1030		51.5			
middle	106	18.7	460		81.3	566		28.3			
poor	90	22.3	314		77.7	404		20.2			
**Outdoor activities**										12.205	0.000
with	163	14.0	1003		86.0	1166		58.4			
without	165	19.9	666		80.1	831		41.6			
**Seeing children**										3.386	0.495
every day	208	16.2	1075		83.8	1283		65.2			
every week	72	17.6	338		82.4	410		20.8			
every month	29	14.9	165		85.1	194		9.9			
every year	15	22.7	51		77.3	66		3.4			
<1 time/year	4	25.0	12		75.0	16		0.8			
**Contact with neighbors**										7.564	0.056
every week	286	15.9	1510		84.1	1796		89.8			
every month	15	19.7	61		80.3	76		3.8			
every year	6	37.5	10		62.5	16		0.8			
almost never	23	20.7	88		79.3	111		5.6			
**Gathering w/relatives**										12.649	0.005
every week	61	13.6	387		86.4	448		22.4			
every month	43	13.8	269		86.2	312		15.6			
every year	145	16.6	727		83.4	872		43.6			
almost never	81	22.1	286		77.9	367		18.4			
**Community activities**										6.254	0.100
every week	40	13.2	262		86.8	302		15.2			
every month	24	15.5	131		84.5	155		7.8			
every year	27	13.0	181		87.0	208		10.4			
almost never	238	17.9	1090		82.1	1328		66.6			
**Volunteer activities**										1.967	0.161
yes	35	13.5	224		86.5	259		13.0			
no	294	17.0	1437		83.0	1731		87.0			

Coming from a poorer area, doing fewer outdoor activities, and taking part in fewer family gatherings were found to have significant positive relationships with hospitalization. Respondents with hypertension (χ^2^ = 7.932, p < 0.05), heart disease (χ^2^ = 41.326, p < 0.05), diabetes (χ^2^ = 4.321, p < 0.05), cataracts (χ^2^ = 9.33, p < 0.05), cerebrovascular disease (χ^2^ = 155.849, p < 0.05), bronchitis (χ^2^ = 34.634, p < 0.05), and gastroenteritis (χ^2^ = 5.958, p < 0.05) were significantly more likely to have been hospitalized in the previous year.

### Logistic regression analysis of hospitalization

The inclusion level was set to p < 0.05 and the exclusion criterion to p > 0.1. Based on these thresholds, all the variables were included in stepwise regression. Table 4 shows the final logistic regression analysis results of each Andersen model predictor of hospitalization rate in the previous year. Older age groups were more likely to have been hospitalized. Those with income from work or savings (model 1: OR 0.511; 95%CI 0.279–0.938, p = 0.030) were less likely to have been hospitalized than those with income from a pension, contrary to the case with outpatient service use. Those living in the outer suburbs were more likely to have been hospitalized (model 1: OR 1.316; 95%CI 0.962–1.8028, p = 0.001). Poor self-reported health status (model 1: OR 3.377; 95%CI 2.234–5.104, p = 0.000), being limited in one or more activity of daily living (ADL) (model 1: OR 2.954; 95%CI 1.388–6.29, p = 0.005), having three types of chronic diseases, and poor regional economic level (model 3: OR 3.429; 95%CI 1.782–6.596, p = 0.000) were positively associated with having been hospitalized.

**Table 4:** Logistic regression analysis of hospitalization.

Variable	Model 1		Model 2		Model 3	
	Sig.	OR (95%CI)	Sig.	OR (95%CI)	Sig.	OR (95%CI)
**Predisposing factors**						
**Age group (years)**						
60–64	ref		ref		ref	
65–69	0.134	1.406 (0.9–2.196)	0.123	1.423 (0.909–2.226)	0.302	1.274 (0.804–2.019)
70–74	0.002*	1.964 (1.286–2.998)	0.001*	2.012 (1.315–3.079)	0.022*	1.676 (1.076–2.611)
75–79	0.000*	2.233 (1.498–3.33)	0.000*	2.22 (1.487–3.316)	0.002 *	1.954 (1.29–2.958)
≥80	0.000*	2.308 (1.529–3.484)	0.000*	2.218 (1.464–3.362)	0.003*	1.925 (1.253–2.957)
**Enabling factors**						
**Source of income**						
pension	ref		ref		ref	
work or savings	0.030*	0.511 (0.279–0.938)	0.026*	0.501 (0.272–0.922)	0.041*	0.521 (0.279–0.974)
family	0.473	0.763 (0.365–1.597)	0.479	0.766 (0.365–1.604)	0.445	0.732 (0.329–1.629)
others	0.026*	0.382 (0.164–0.892)	0.028*	0.387 (0.166–0.902)	0.191	0.566 (0.241–1.329)
**Location**						
city center	ref		ref		ref	
inner suburbs	0.086	1.316 (0.962–1.802)	0.001*	2.774 (1.515–5.08)	0.000*	3.527 (1.852–6.719)
outer suburbs	0.000*	2.582 (1.751–3.808)	0.000*	5.665 (2.95–10.877)	0.000*	6.024 (3.013–12.045)
**Need factors**						
**Self-reported health status**						
good	ref		ref		ref	
normal	0.000*	1.76 (1.282–2.416)	0.001*	1.752 (1.273–2.412)	0.006*	1.583 (1.138–2.202)
poor	0.000*	3.377 (2.234–5.104)	0.000*	3.211 (2.116–4.873)	0.000*	2.456 (1.578–3.822)
**Feeling lonely**						
never	ref		ref		ref	
sometimes	0.071	1.389 (0.973–1.982)	0.111	1.339 (0.935–1.918)	0.336	1.201 (0.827–1.746)
always	0.162	0.585 (0.276–1.241)	0.121	0.55 (0.258–1.171)	0.133	0.549 (0.251–1.2)
**ADLs** (no vs. yes)	0.005*	2.954 (1.388–6.29)	0.006*	2.94 (1.364–6.34)	0.071	2.143 (0.937–4.901)
**Physical health change**						
better	ref		ref		ref	
unchanged	0.000 *	0.21 (0.128–0.344)	0.000*	0.213 (0.129–0.349)	0.000*	0.22 (0.133–0.365)
worse	0.002*	0.457 (0.275–0.758)	0.002*	0.451 (0.27–0.752)	0.001*	0.423 (0.251–0.712)
unstable	0.143	0.479 (0.179–1.284)	0.165	0.493 (0.181–1.338)	0.117	0.441 (0.158–1.227)
**Disease status**						
**Diabetes** (yes vs. no)					0.692	0.926 (0.633–1.355)
**Heart diseases** (yes vs. no)					0.008*	1.535 (1.12–2.104)
**Cerebrovascular disease** (yes vs. no)					0.000*	4.572 (3.029–6.901)
**Bronchitis** (yes vs. no)					0.009*	1.886 (1.173–3.031)
**Contextual Factors**						
**Regional economic level**						
good			ref		ref	
poor			0.003*	2.539 (1.374–4.694)	0.000*	3.429 (1.782–6.596)
**Outdoor activities** (no vs. yes)			0.065	1.306 (0.983–1.736)	0.107	1.273 (0.949–1.708)
**Chi-squared**	207.205				219.544			287.463
**df**	17		19		23	
**Sig.**	0.000		0.000		0.000	
**-2Log Likelihood**	1421.322		1408.983		1341.064	

*p < 0.05;

OR: odds ratio; CI: confidence interval

Next, the -2Log Likelihood (Model 1) was 1421.322. After adjusting for the predictors in Model 1, adding the contextual factors, the -2Log Likelihood for Model 2 was 1408.983. After adjusting for the predictors in Model 2, having diseases predicted hospitalization, and the -2 Log Likelihood for Model 3 was 1341.064.

## Discussion

This study improves our understanding of factors that influence use of healthcare services by older people in Shanghai and other Chinese cities, especially factors related to disease status and contextual factors, which have only rarely been considered previously.

### Predisposing factors

We observed that predisposing factors including age, gender, pension income level, source of income, and marital status were statistically associated with utilization of health services in univariate analysis. Meanwhile, age contributed significantly to variance in utilization of hospitalization in logistic regression analysis.

There are some controversies around findings related to gender. Some studies have suggested that women are more likely to have used outpatient services in the previous two weeks than men [36] and that this might be related to women’s physical and psychological characteristics, since they more often belong to vulnerable groups [37]. Some studies have found that men are more likely to delay treatment than women because of social and behavioral factors [26]; however, other studies suggested the opposite [38]. This study found that the female outpatient visit rate in the previous two weeks was higher than that of men.

Older individuals tend to have more need for healthcare because they usually have more comorbid conditions [39, 40] and suffer from more adverse effects of treatment [41]. This study also found that with increasing age, the annual admission rate increased, which is consistent with prior research [42].

Previous investigations of living conditions and education have shown conflicting results. Some studies have indicated that older people living alone are more likely to be admitted to hospital than those living with an informal caregiver [43]. Education was positively and significantly related to use of outpatient services in some previous research [3, 44, 45]; however, other studies [46, 47] showed that older people with a lower educational level are more likely to visit their general practitioner. The present study reported no links between healthcare use and either living conditions or education. Women and/or in older people should be a key target groups for health interventions.

### Enabling factors

The observations that source of income [48–51] and regional economic development were significantly related to health service utilization among older people are consistent with earlier research [52, 53]. Compared with those whose income came from pensions, work, or family, those whose income came from friends or social relief had visited healthcare services more in the previous two weeks but been hospitalized less in the previous year. This is probably because those relying on friends or social relief cannot afford expensive hospital care and are therefore more likely to use outpatient services.

Higher health service utilization was seen among those living further away from the city center. In general, older people living nearer to the city center tend to live in nursing or residential homes because they have less access to family care and more of this support infrastructure because of greater local economic development. This finding may be the result of the stratified cluster sampling used in this study, because those living in such institutions, who usually need more healthcare, were excluded from our sample. To promote equitable healthcare utilization among older people living in the community, relevant departments and agencies should provide sufficient care for those living in outer suburbs and those whose incomes comes from friends or social relief, as these groups tend to use health services more.

### Need factors

Previous research has generally found that health service use is mainly associated with need variables [36, 54]. This study similarly observed that self-reported health status, general level of life satisfaction, physical health change, feelings of loneliness, and limitations in ADLs were significantly, positively related to health service utilization.

Self-reported health status reflects the feelings, ideas, and beliefs of individuals about their health [55, 56]. An individual’s decision to use health services is the result of a complex interaction of factors relating to their health or self-perceived health status and to the availability of healthcare [36]. Consistent with other research, we found that older people with poor self-reported health had significantly higher odds of using both outpatient and inpatient services (2.469 and 2.456 times that of healthier individuals, respectively). Older people whose health status worsens tend to use more outpatient services but to be hospitalized less. Meanwhile, older people who are limited in ADLs have higher odds of being hospitalized than those without any such limitations. Elderly people with higher anxiety, depression, and/or concerns about their health and life have less capacity to resist disease and so also tend to use more healthcare services. However, healthcare utilization was not associated with sensation disorders, again consistent with previous studies [36].

### Disease status

We also looked at the effect of special need factors—various chronic diseases on healthcare service utilization. The seven most prevalent diseases—hypertension, heart disease, diabetes, cataracts, cerebrovascular disease, bronchitis, and gastroenteritis—were added into the model. The study found that older people with heart disease and gastroenteritis use more outpatient services, while those with heart disease, cerebrovascular disease, and bronchitis tend to be hospitalized more. Those with hypertension or diabetes use fewer healthcare services, probably because these diseases are more stable and can be controlled through medication and other self-treatments.

Diseases such as heart disease, cataracts, and cerebrovascular disease have a longer course, and easily lead to complications and morbidity. Health education on age-related diseases, especially chronic diseases, should be carried out in the community to help people prevent and control these diseases, maintain a stable state of health, and improve their quality of life.

### Contextual factors

Contextual factors are another important, although often neglected group of factors affecting healthcare utilization [21, 23, 24]. We found that older people who engage in volunteering tend to use more outpatient services. This might be because the government has developed many community health services and promoted their utilization. Living in a poorer region and participating fewer outdoor activities were also positively related to higher healthcare use. However, compared with the poorer medical conditions in outer suburbs, richer areas often have better medical services and more skilled personnel, which may improve disease prevention, management, and prognosis. More attention should also be paid to older people’s psychological needs, such as for psychological guidance and comfort, especially among those in poor physical condition and/or those who do not spend time in outdoor activities. The key to improving healthcare utilization is to improve older people’s social environment, through increased social support and availability of activities near home. A wider range of healthy activities could be arranged within the community to promote older people’s mental and physical health and strengthen their psychological self-adjustment.

## Conclusions

The results showed the impact of economic status, health status, demographic and social characteristics, and other factors on the health service utilization of elderly people living in the community in Shanghai. Need variables in the Andersen model, including self-reported health status, life satisfaction, physical health change, and disease status, were the strongest factors influencing health service use, consistent with previous research [14, 20]. Contextual factors, especially regional economic level and volunteer activities, also contributed to it.

### Limitations

This study has several limitations. The first is its cross-sectional design. The method of investigation was a self-reported household survey, which may have led to recall bias and affected the accuracy of the survey results. A longitudinal study would be helpful in the future, to collect data through long-term continuous tracking and provide time-series data to improve understanding. Additionally, while healthcare services include primary care [57], preventive health services [58], outpatient services [59], ambulatory care [60] and hospital inpatient services [61], we focused only on use of outpatient and inpatient services. In addition, only people aged above 60 living in the community in Shanghai were sampled, while those living in nursing homes or pension agencies were excluded even though they may have more need for healthcare services. Their situation should be explored in a further study.

## Supporting information

S1 File Outpatient database. Database used for univariate analysis and logistic regression analysis of outpatient health service utilization.
(XLSX)

S2 File Inpatient database. Database used for univariate analysis and logistic regression analysis of hospitalization.
(XLSX)
